# Mutagenesis of *FT* genes in early flowering kiwifruit suggests diverse roles in regulation of growth and flowering

**DOI:** 10.1093/plphys/kiaf473

**Published:** 2025-11-06

**Authors:** Erika Varkonyi-Gasic, Tianchi Wang, Dinum Herath, Charlotte Voogd, Andrew C Allan, Joanna Putterill

**Affiliations:** The New Zealand Institute for Plant & Food Research Limited (Plant & Food Research) Mt Albert, Auckland 1025, New Zealand; The New Zealand Institute for Plant & Food Research Limited (Plant & Food Research) Mt Albert, Auckland 1025, New Zealand; The New Zealand Institute for Plant & Food Research Limited (Plant & Food Research) Mt Albert, Auckland 1025, New Zealand; The New Zealand Institute for Plant & Food Research Limited (Plant & Food Research) Mt Albert, Auckland 1025, New Zealand; The New Zealand Institute for Plant & Food Research Limited (Plant & Food Research) Mt Albert, Auckland 1025, New Zealand; School of Biological Sciences, University of Auckland, Auckland 1010, New Zealand; School of Biological Sciences, University of Auckland, Auckland 1010, New Zealand

## Abstract

Kiwifruit *FT* primarily controls the ability of shoots to grow, *FT1* regulates the timing of terminal flowering, while *FT2* and *YFT* have no effect on flowering time in the early flowering kiwifruit.

Dear Editor,

Kiwifruit (*Actinidia chinensis*) produce a high-value fruit crop (Chamberlain 2023), yet reproductive challenges remain including a long nonflowering juvenile phase and a temperate phenology where warming winters induced by climate change reduce effective spring budbreak and flowering (Richardson and Varkonyi-Gasic 2023). *FLOWERING LOCUS T*-like (*FT*-like) genes typically encode mobile florigens that function as powerful activators of flowering, while the related *CENTRORADIALIS*-like (*CEN*-like) genes function as flowering repressors (Wickland and Hanzawa 2015). Consistent with this, we previously demonstrated that mutagenesis of kiwifruit *CEN* or *CEN4* was sufficient for precocious and continuous terminal flowering; even earlier flowering and shorter internodes were seen in the very compact *cen cen4* double mutants (Varkonyi-Gasic et al. 2019). In addition, overexpression of kiwifruit *FT*-like genes *FT*, *FT1* or *FT2* promoted in vitro and terminal flowering in kiwifruit (Voogd et al. 2017; Moss et al. 2018; Herath et al. 2023), indicating they functioned as floral activators. However, *FT* gene expression patterns (Varkonyi-Gasic et al. 2013; Voogd et al. 2017) suggested possible additional or alternative functions. Long juvenility makes it very time consuming and challenging to study the role of *FT* paralogs by mutagenesis in woody perennials (Putterill and Varkonyi-Gasic 2016; Cardi et al. 2023) and this has not been accomplished yet in wild type (WT) kiwifruit. Therefore here, we use early flowering *cen4* and *cen cen4* mutants to investigate the roles of kiwifruit *FT*-like genes in precocious terminal flowering. Unexpectedly, mutagenesis of *FT* did not abolish the early flowering of *cen4* and *cen cen4* plants. Instead, arrest of both growth and lateral shoot development indicated that *FT* primarily controls the ability of shoots to grow. Mutagenesis of *FT1* moderately delayed terminal flowering in *cen cen4 ft1* mutants but *cen4 ft1* mutants did not flower, suggesting that terminal flowering may be controlled by both flowering activator FT1 and CEN flowering repressors. Editing of *FT2* and a fourth, male kiwifruit-specific *YFT* (Akagi et al. 2018, 2023) did not prevent early flowering in hermaphrodite kiwifruit *cen4 sygl* mutants (Varkonyi-Gasic et al. 2021).

In WT kiwifruit *FT* is expressed in source leaves, consistent with the ability to act as a mobile signal; in axillary buds at the time of re-establishment of spring growth, coinciding with flower development; and in flower buds and fruit (Supplementary Fig. S1). On the other hand, *FT1* accumulates in shoot tips (Supplementary Fig. S1). Despite a largely different architecture to WT, comparable expression of *FT* and *FT1* were detected in *cen cen4* and *cen4* early flowering lines (Supplementary Fig. S2). These lines were then used to study the roles of *FT* and *FT1*, using CRISPR-Cas9-mediated mutagenesis (Fig. 1, A and B; Supplementary Fig. S3 and Tables S1 to S3). The possible contribution of two other *FT*-like genes, *FT2* and the sex-linked *YFT* to early flowering could not be formally excluded, although only low levels of their transcripts were detected in WT plants (Akagi et al. 2018) (Supplementary Fig. S1) and we could not detect *FT2* expression in the *cen cen4* and *cen4* kiwifruit samples. Therefore, constructs targeting *FT2* and *YFT* were also used (Fig. 1A; Supplementary Table S1) and edited lines were generated (Supplementary Tables S2 and S3).

**Figure 1.**
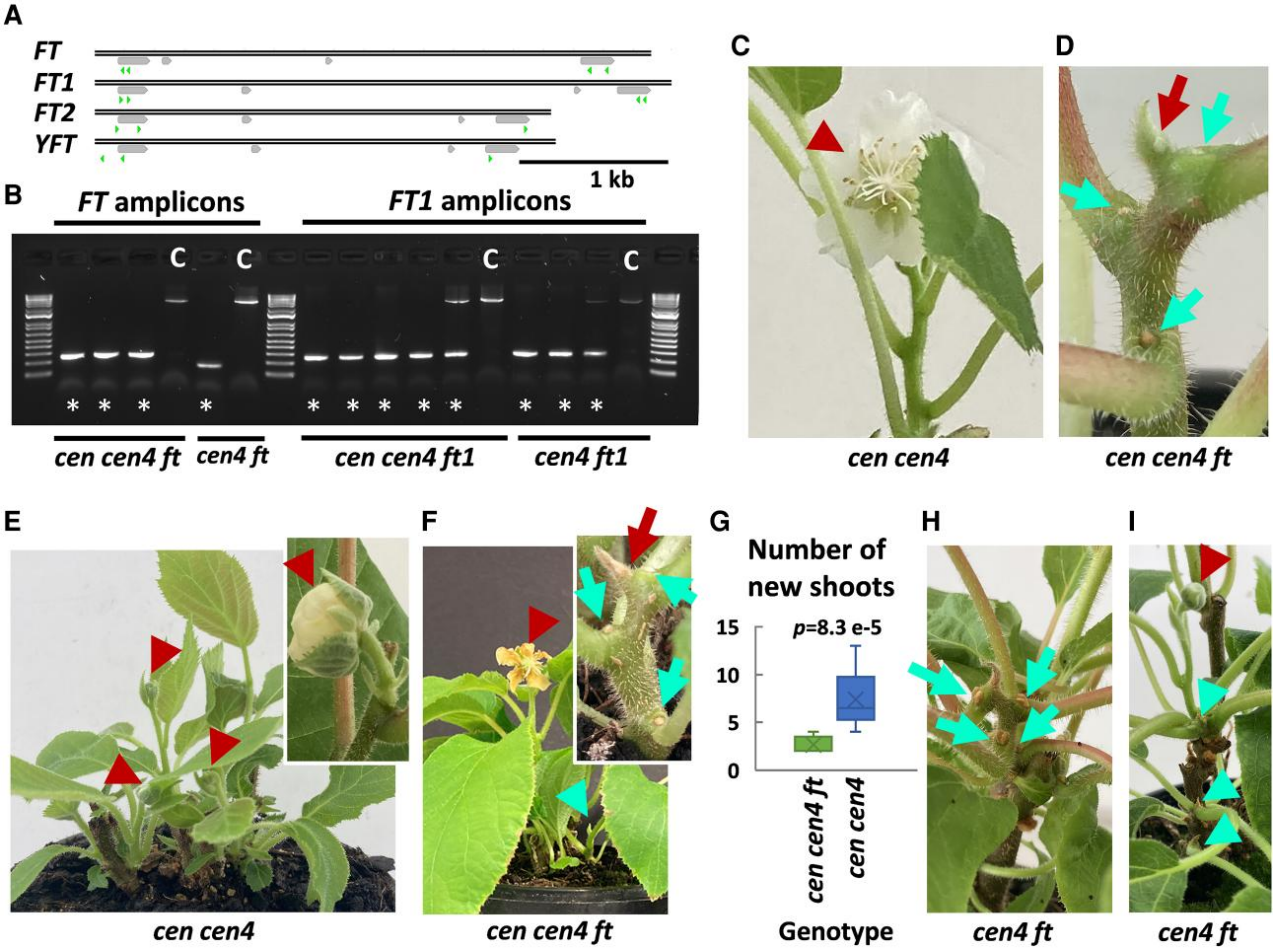
Mutagenesis of *FT* (*ft*) does not abolish terminal flowering in early flowering *cen cen4* kiwifruit but induces bud set. **A)** Schematics demonstrating the gene structure of four kiwifruit *FT*-like genes with exons presented as gray boxes and target sequences for CRISPR-Cas9-mediated mutagenesis (Supplementary Table S1) in green. **B)** Gel electrophoresis of amplicons from *ft* and *ft1* lines chosen for analysis (asterisks) and nonedited controls (c), demonstrating large deletions between target sequences in both or one of the alleles. Genotyping by sequencing of cloned PCR fragments and whole genome sequencing of a subset of lines confirmed biallelic edits (Supplementary Fig. S3 and Table S3). **C** and **D)** Mutations in *FT* did not prevent terminal flowering but induced flower abortion and bud set in *cen cen4 ft* kiwifruit. **E)** Pruning of *cen cen4* plant gives rise to new shoots with terminal flowers. Inset shows a typical short shoot with a terminal flower. **F)** Emergence of a floral shoot and aborted shoot in a *cen cen4 ft* line after pruning. Inset shows a close-up of a typical aborted shoot after pruning. **G)** Reduced number of new shoots in *cen cen4 ft* line. Three *cen cen4 ft* and four *cen cen4* lines were pruned and the number of newly emerging shoots was scored. The whiskers indicate the minimum and maximum, the bottom and top of the box are the 25th and 75th percentiles, the line inside the box is the 50th percentile (median), and the cross represents the mean. The *t*-test *P*-value (<0.05) demonstrating a statistically significant difference is given above. **H** and **I)** Growth arrest and emergence of floral buds and aborted shoots in a *cen4 ft* mutant after pruning. **C-F**, **H**, and **I)** Flowers and flower buds are indicated with red arrowheads, red arrows mark the aborted flowers, axillary bud set is indicated with teal arrows and aborting shoots with teal arrowheads.

In triple *cen cen4 ft* mutants we observed early termination of growth and arrest of flower development on the primary shoot (Fig. 1, C and D). Growth and terminal flowering could be re-established by pruning, but a large difference in the numbers of shoots and their growth and flowering patterns from those of *cen cen4* controls was seen (Fig. 1, E to G, Supplementary Table S4). Pruning of *cen cen4* control lines resulted in outgrowth of multiple shoots from lateral buds and buds at the base of the stem, each terminating in a flower (Fig. 1E). In contrast, in the *cen cen4 ft* lines, a new shoot with a terminal flower was able to develop after pruning, but development of new shoots and flowers was aborted (Fig. 1F). Therefore, mutagenesis of *FT* in *cen cen4* lines did not abolish flowering; rather it induced axillary bud set and prevented shoot outgrowth, resulting in arrested flower development and reduced number of shoots (Fig. 1G). Axillary bud set (Fig. 1H) and premature termination of new shoot growth after pruning was also observed in double *cen4 ft* mutants, however they also retained the ability to flower (Fig. 1I).

In contrast to *FT*, *FT1* accumulated in the distal end of the shoot of WT, *cen cen4* and *cen4* mutant kiwifruit (Supplementary Figs. S1 and S2), suggesting that *FT1* may contribute to terminal flowering of these mutants. Triple *cen cen4 ft1* mutants showed moderately delayed flowering (Fig. 2A). This increased the number of nodes at which terminal flowering occurred, without a significant effect on the number of shoots emerging after pruning (Fig. 2, B and C; Supplementary Table S5). In contrast, *FT1* mutagenesis in *cen4* resulted in no flowering in *cen4 ft1* lines over the duration of the experiment (Fig. 2, D and E). Conversely, mutagenesis of *FT2* and the male-specific *YFT* (Supplementary Table S2) did not prevent early terminal flowering of the *cen4 sygl* mutant (Fig. 2, F to H; Supplementary Table S6), a male kiwifruit previously converted to hermaphrodite using gene editing of the sex-determining *SYGL* gene (Varkonyi-Gasic et al. 2021).

**Figure 2.**
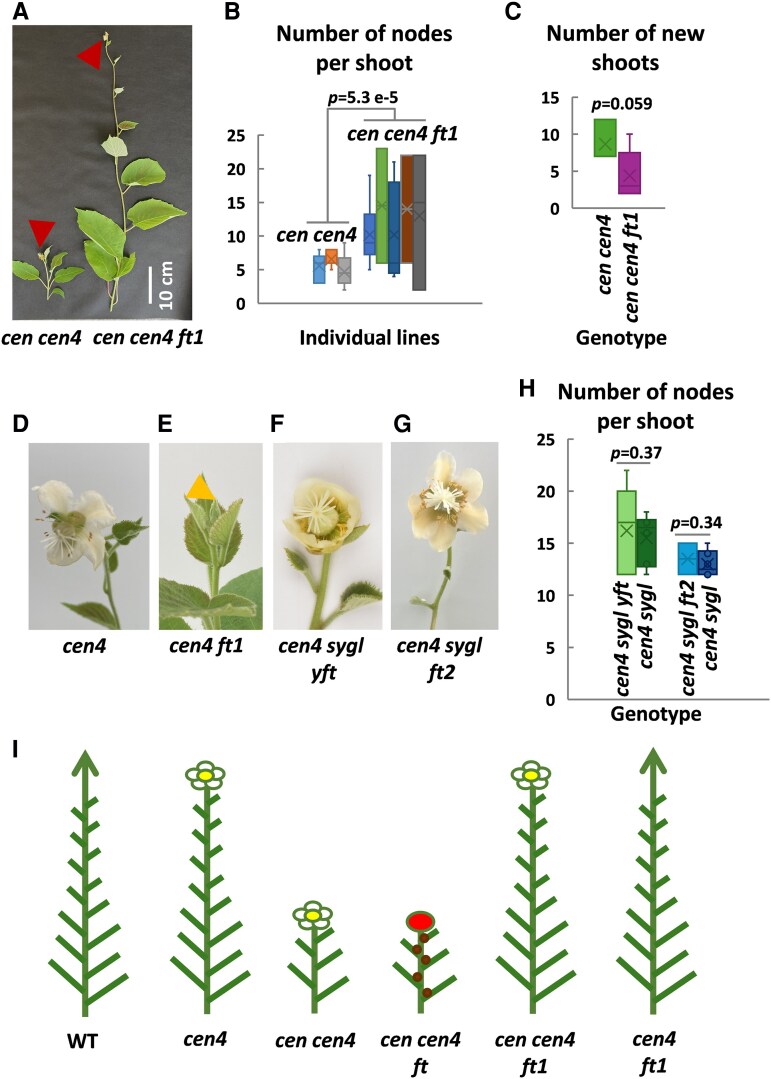
Mutagenesis of *FT1* (*ft1*) delays termination but does not abolish precocious terminal flowering in *cen cen4* plants. **A** and **B)** Mutations in *FT1* delay terminal flowering in *cen cen4 ft1* plants, resulting in longer primary shoots **(A)** and increased number of nodes per shoot after pruning **(B)**, measured on multiple shoots emerging from the base of three *cen cen4* and five *cen cen4 ft1* lines. Red arrowheads indicate terminal flowers. The whiskers indicate the minimum and maximum, the bottom and top of the box are the 25th and 75th percentiles, the line inside the box is the 50th percentile (median), and the cross represents the mean. The *t*-test *P*-value (< 0.05) demonstrating a statistically significant difference is given above. **C)** The number of new shoots emerging from the base of three *cen cen4* and five *cen cen4 ft1* lines described in **(B)**. The box plots are as described **(B)** and the *t*-test *P*-value indicated above (> 0.05) suggests no significant difference. **D** and **E)** No terminal flowering in *cen4 ft1* mutant. Orange arrowhead marks the vegetative shoot tip. **F–H)** Mutagenesis of *YFT* (*yft*) or *FT2* (*ft2*) did not affect flower development **(F** and **G)** or significantly (*t*-test *P*-values > 0.05) delay flowering as measured by node number **(H)** in hermaphrodite *cen4 sygl* kiwifruit. Five *cen4 sygl yft* and two *cen4 sygl ft2* lines were studied alongside six *cen4 sygl* controls each. The box plots are as described above **(B)**. **I)** Schematics summarizing the key findings. Editing of one or both *CENTRORADIALIS*-like genes *CEN* and *CEN4* converts the woody perennial vine kiwifruit with long juvenility (WT) into compact (*cen cen4*) and small vine (*cen4*) plants with early terminal flowering. In these mutants, absence of *FT* results in aborted growth and terminal flower development, represented by brown circles depicting bud set and a red circle representing the arrested terminal flower. This implies that *FT* primarily controls growth. Absence of *FT1* resulted in prolonged vegetative growth and delayed terminal flowering in *cen cen4 ft1* and no terminal flowering in *cen4 ft1* mutants. This shows that terminal flowering is regulated by flowering repressors CEN and CEN4 and flowering activator FT1. Arrows indicate vegetative shoot tips and flower drawings represent terminal flowers.

Our results imply conservation of *FT*-like roles in phenology, growth and architecture (Fig. 2I), separate from floral induction, in temperate woody perennial species as reported for *Populus* (Klocko et al. 2018; André et al. 2022; Sheng et al. 2023). On the other hand, kiwifruit terminal flowering is regulated by flowering CEN-like repressors CEN and CEN4 and flowering activator FT1 (Fig. 2I). It remains to be determined if floral induction and axillary flower development in basal nodes typical of WT kiwifruit (Supplementary Fig. S2A) require any FT-like activity, and which of the kiwifruit *FT*-like genes may be involved. Ultimately, uncovering the precise role and interactions of different *FT*-like and *CEN*-like genes in a range of perennial species will be key to understanding how flowering and growth are fine-tuned in temperate woody plants, opening opportunities for targeted breeding strategies and improved horticultural management in the context of climate change.

## Supplementary Material

kiaf473_Supplementary_Data

## Data Availability

The data presented in this study are available in the Supplementary Material.

## Supplementary data

The following materials are available in the online version of this article.

**Supplementary Figure S1.** Expression of *FT* genes in WT kiwifruit

**Supplementary Figure S2.** Kiwifruit plant architecture and *FT* gene expression in *cen4* and *cen cen4* mutants

**Supplementary Figure S3.** CRISPR-Cas9-mediated gene editing of kiwifruit *FT* genes

**Supplementary Table S1.** CRISPR-Cas9 gene editing experimental design

**Supplementary Table S2.** Primer sequences used for amplifications and sequencing for genotyping CRISPR-Cas9 genome edited lines

**Supplementary Table S3.** Genotyping CRISPR-Cas9 genome edited lines

**Supplementary Table S4.** Scoring the number of new shoots after pruning (presented in Fig. 1G)

**Supplementary Table S5.** Flowering time scored as a number of nodes on floral shoots and number of new floral shoots (presented in Fig. 2B, C)

**Supplementary Table S6.** Flowering time scored as a number of nodes on the primary (longest) floral shoot (presented in Fig. 2H)
