# Supplementary material for: Mutagenesis of FT genes in early flowering kiwifruit suggests diverse roles in regulation of growth and flowering
Source: Plant Physiol. 2025 Nov 6;199(3):kiaf473. doi: 10.1093/plphys/kiaf473 (PMC12596246; doi:10.1093/plphys/kiaf473)
Supplement: kiaf473_Supplementary_Data [file kiaf473_supplementary_data.zip › Supplementary data_revision_clean.pdf]

SUPPLEMENTARY DATA

Mutagenesis of *FT* genes in early flowering kiwifruit suggests diverse roles in regulation of growth and flowering

Erika Varkonyi-Gasic[1]*, Tianchi Wang[1], Dinum Herath[1], Charlotte Voogd[1], Andrew C Allan[1,2], Joanna Putterill[2]

SUPPLEMENTARY MATERIALS AND METHODS

Plant material and growth conditions

Plant material from kiwifruit *Actinidia chinensis* Planch. var. *chinensis* 'Hort16A' early-flowering *cen cen4* and *cen4* female plants (Varkonyi-Gasic et al., 2019) and *A. chinensis* 'Bruce' *sygl cen4* early-flowering XY and YY hermaphrodites (Varkonyi-Gasic et al., 2021) was obtained from in vitro collections held at Plant & Food Research, Auckland, New Zealand. This early flowering material was used for further transformation to generate edits in kiwifruit *FT*, *FT1*, *FT2* and *YFT*. Rooted transgenic plants were potted up using standard potting mix, established in the growth room with controlled conditions (22°C, 16 h/8 h light/dark) for three weeks, then grown in glasshouse growth rooms (temperature min 18°C/max 30°C night/day, 14 h/10 h light/dark).

RNA extraction and expression studies

Expression in wild-type (WT) kiwifruit plant organs, terminal and axillary buds was according to described RNA-seq experiments (Brian et al., 2021; Voogd et al., 2022). For quantifications of expression in early flowering *cen cen4* and *cen4* lines, total RNA was isolated using the Spectrum Plant Total RNA Kit (Sigma-Aldrich, St. Louis, MA, USA) and reverse transcribed using the QuantiTect Reverse Transcription Kit (Qiagen) following the manufacturers' instructions. Amplification primers and quantification using real-time PCR were according to Varkonyi-Gasic et al. (2013) and Voogd et al. (2017). Expression normalized to *Actinidia ACTIN* was presented as mean ± SE of three biological replicates (samples collected from three individual lines).

Vectors and constructs

For CRISPR-Cas9-mediated mutagenesis, target sequences (Table S1) were identified using the Geneious 10.0.9 (https://www.geneious.com) CRISPR selection tool and criteria described by Doench et al. (2014). The constructs were designed to contain a polycistronic tRNA-sgRNA cassette with up to four gene-specific sgRNA sequences, placed under the control of the Arabidopsis U6-26 promoter and flanked by Gateway™ recombination sites were synthesized (Genewiz, South Plainfield, NJ, USA; www.genewiz.com), then recombined with the destination vector pDE-KRS-HYG (Herath et al., 2022), which expresses *Cas9* from the *35S* promoter and contains the hygromycin resistance cassette. All constructs were introduced by electroporation into *Agrobacterium tumefaciens* strain EHA105. *Agrobacterium*-mediated transformation and hygromycin selection were as previously described (Herath et al., 2022). At least 10 independent lines were established for each construct and these were analysed in the T0 generation.

Genomic DNA extraction and genotyping

Genomic DNA was extracted from leaf tissue using the DNeasy plant Mini Kit (Qiagen, Hilden, Germany) as per the manufacturer's instruction. PCR amplification was performed using iProof High-Fidelity DNA Polymerase (Bio-Rad Laboratories, Hercules, CA, USA) with kiwifruit *FT*, *FT1*, *FT2* and *YFT* gene-specific oligonucleotide primers (Table S2) and previously described *CEN* and *CEN4* oligonucleotide primers (Varkonyi-Gasic et al., 2019). The PCR amplification consisted of initial denaturation at 98°C (2 min), followed by 36 cycles of denaturation at 98°C (10 s), annealing at 57°C (15 s), and extension at 72°C (30 s/kb), followed by a final extension at 72°C (5 min). Amplification products were analysed by agarose gel electrophoresis. Where possible, lines with large deletions in target genes identified by gel electrophoresis were chosen for further analysis. Subsequently, the amplification products were purified using the DNA Clean & Concentrate Kit (Zymo Research, Irvine, CA, USA) and sent for sequence analysis (Macrogen, Seoul, Republic of Korea), or cloned into pJET1.2/blunt cloning vector supplied in the CloneJET PCR cloning kit (Thermo Fisher Scientific, Waltham, MA, USA) prior to sequencing at least 5 clones. Sequences were compared with appropriate annotated sequences in the reference *A. chinensis* Red5 genome (Pilkington et al., 2018) using ClustalW within Geneious 10.0.9 (https://www.geneious.com). A subset of lines was chosen for further sequence

verification using whole genome sequencing, performed at Annoroad Gene Technology Limited (Beijing, China) on the DNBSEQ-T7 platform (MGI, Shenzhen, China) with paired-end read length of 150 bp and 20X read coverage. The reads were mapped to the reference *A. chinensis* Red5 genome (Pilkington et al., 2018) using BWA (Li and Durbin, 2009) (v0.7.18) and sorted using SamTools (v1.20) (http://www.htslib.org/doc/1.20/samtools.html). The reads mapped to genes of interest were interrogated for edits by comparison to WT sequences in Geneious Prime 2025.0.2 (https://www.geneious.com).

Plant phenotyping

For phenotyping analyses, three *cen4 cen ft*, one *cen4 ft*, five *cen4 cen ft1*, three *cen4 ft1* and two *cen4 sygl ft2* lines with confirmed bi-allelic edits in indicated genes, as well as five *cen4 sygl yft* lines with hemizygous edits in the sex-linked *YFT* gene were chosen and compared to the minimum of three counterpart lines (Table S1) with no mutations in *FT* genes. Plants were monitored daily for a minimum of four months from establishment in the glasshouse, at which stage all the early flowering lines produced shoots with terminal flowers. As reported before, the compact *cen cen4* lines were flowering after developing shoots with 6-8 leaves and the early flowering *cen4* vines flowered with 12-15 leaves (Varkonyi-Gasic et al., 2019). Increased number of nodes at which terminal flowering occurred when compared to counterparts with no *FT* gene edits was noted as delayed flowering. Plants were considered non-flowering after monitoring for a minimum of six months and after allowing their longest shoot to grow >40 nodes. Plants that set buds and arrested the development of their first terminal flower were recorded as having bud set. To stimulate growth and determine the number of new shoots and the number of nodes on floral shoots, pruning was performed. Shoot tip and upper nodes were removed, leaving 2–4 basal nodes, and the total number of emerging shoots from lateral buds and the base of the stem was counted over a period of up to two months.

SUPPLEMENTARY FIGURES

Supplementary Figure S1

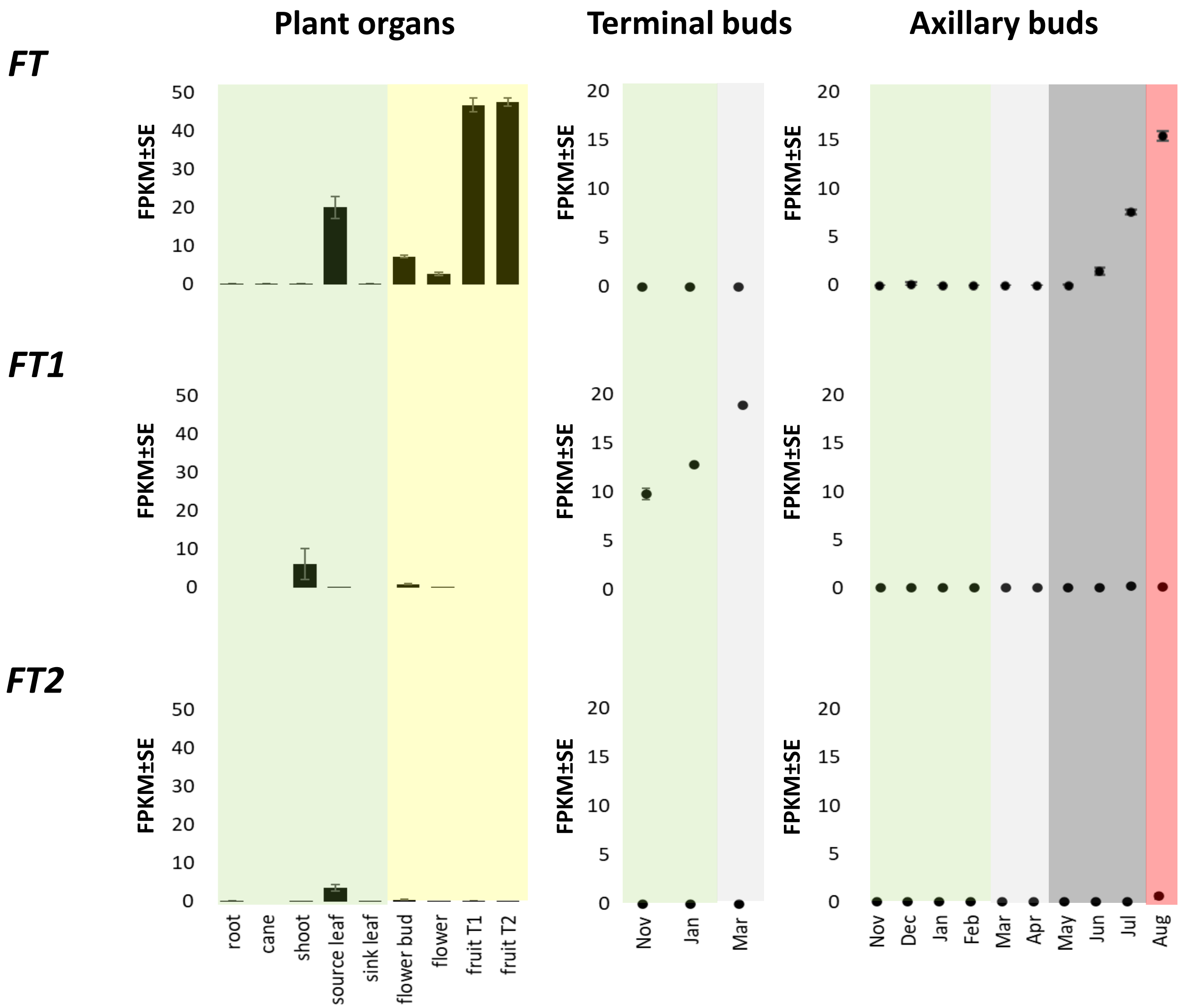

Supplementary Figure S1. Expression of *FT*-like genes in WT kiwifruit. Expression in kiwifruit plant organs, terminal buds during the growing season and shoot axillary buds collected at monthly intervals during the growing and dormancy cycle was determined previously (Brian et al., 2021; Voogd et al., 2021) and presented as mean FPKM ± SE of three biological replicates. Green and yellow shading indicate active vegetative and reproductive growth, respectively; light and dark grey correspond to growth cessation and dormancy, respectively; pink indicates re-establishment of shoot growth in spring, concomitant with flower development.

Supplementary Figure S2

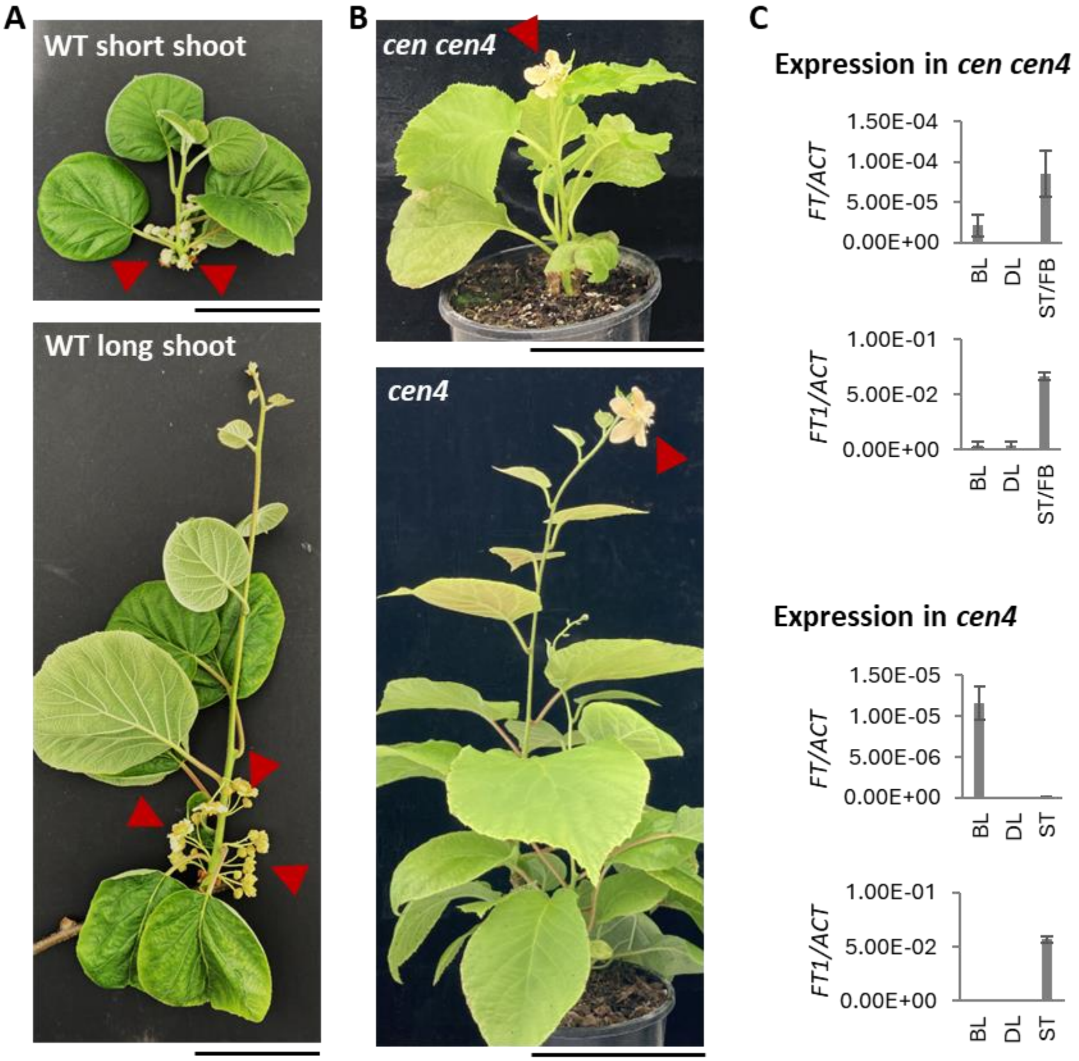

Supplementary Figure S2. Kiwifruit plant architecture and *FT*-like gene expression in *cen4* and *cen cen4* mutants. A. Kiwifruit produce short and long shoots (Foster et al., 2007) with axillary inflorescences (red arrowheads) in basal nodes of mature wild-type (WT) plants. B. Editing of one or both *CENTRORADIALIS*-like genes *CEN* and *CEN4* results in early flowering compact (*cen cen4*) and small vine (*cen4*) kiwifruit plants with terminal flowers (red arrowheads). Both genotypes exhibit continuous flowering and a bushy growth habit, with new shoots arising from buds at the base of the stem, showing progressively faster terminal flowering at reduced node numbers. This is especially evident in compact lines, where hard pruning is needed to promote new shoot emergence and successive

cycles of growth and flowering. A, B. Bars represent 10 cm. C. Expression of kiwifruit *FT* and *FT1* in early flowering mutants, detected by RT-qPCR and presented as mean ± SE of three biological replicates, relative to *ACTIN* (*ACT*). *FT2* was not detected in these samples. Sampling of basal leaves (BL), distal leaves (DL) and shoot tips (ST) was performed when floral buds (FB) were visible in *cen cen4* but not in *cen4* lines.

Supplementary Figure S3

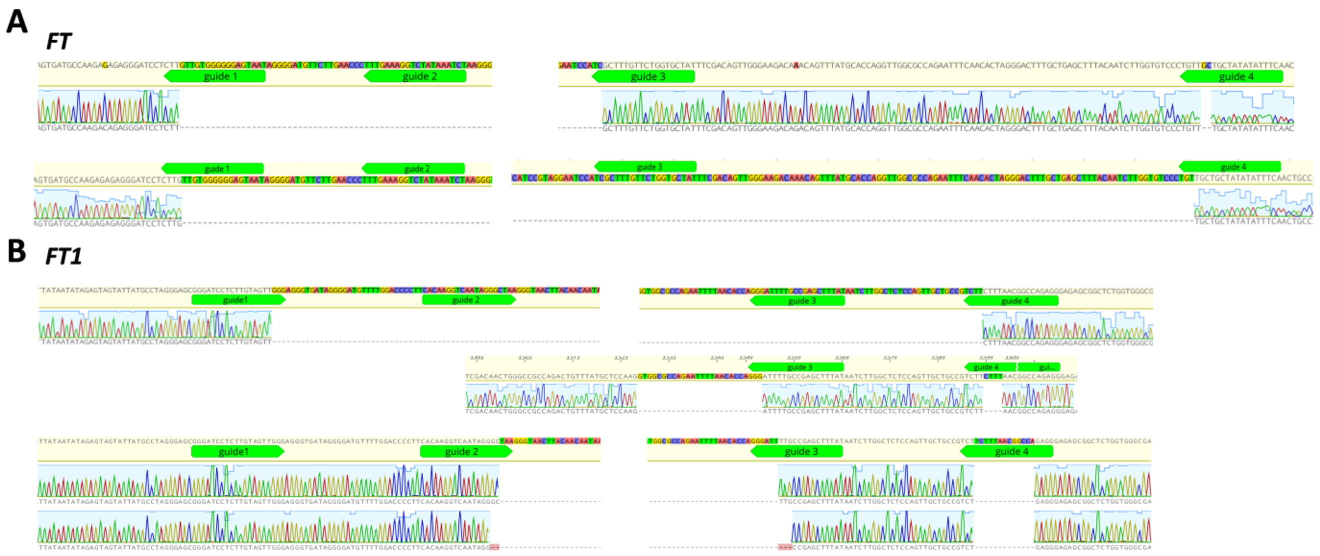

Supplementary Figure S3. CRISPR-Cas9-mediated gene editing of kiwifruit *FT*-like genes. Sequence analysis of *FT* amplicons confirming large deletions between target sequences (A) and large and small deletions between and within target sequences in *FT1* (B). The reference sequences are presented above the sequencing chromatograms, and the guides are indicated with green block arrows below the reference target regions.

REFERENCES

Brian L, Warren B, McAtee P, Rodrigues J, Nieuwenhuizen N, Pasha A, David KM, Richardson A, Provart NJ, Allan AC, Varkonyi-Gasic E, Schaffer RJ (2021) A gene expression atlas for kiwifruit (*Actinidia chinensis*) and network analysis of transcription factors. BMC Plant Biology **21:** 121

Doench JG, Hartenian E, Graham DB, Tothova Z, Hegde M, Smith I, Sullender M, Ebert BL, Xavier RJ, Root DE (2014) Rational design of highly active sgRNAs for CRISPR-Cas9-mediated gene inactivation. Nat Biotechnol **32:** 1262-1267

Foster TM, Seleznyova AN, Barnett AM (2007) Independent control of organogenesis and shoot tip abortion are key factors to developmental plasticity in kiwifruit (*Actinidia*). Ann Bot **100:** 471-481

Herath D, Voogd C, Mayo-Smith M, Yang B, Allan AC, Putterill J, Varkonyi-Gasic E (2022) CRISPR-Cas9-mediated mutagenesis of kiwifruit *BFT* genes results in an evergrowing but not early flowering phenotype. Plant Biotechnology Journal **20:** 2064-2076

Li H, Durbin R (2009) Fast and accurate short read alignment with Burrows–Wheeler transform. Bioinformatics **25:** 1754-1760

Pilkington SM, Crowhurst R, Hilario E, Nardozza S, Fraser L, Peng Y, Gunaseelan K, Simpson R, Tahir J, Deroles SC, Templeton K, Luo Z, Davy M, Cheng C, McNeilage M, Scaglione D, Liu Y, Zhang Q, Datson P, De Silva N, Gardiner SE, Bassett H, Chagné D, McCallum J, Dzierzon H, Deng C, Wang Y-Y, Barron L, Manako K, Bowen J, Foster TM, Erridge ZA, Tiffin H, Waite CN, Davies KM, Grierson EP, Laing WA, Kirk R, Chen X, Wood M, Montefiori M, Brummell DA, Schwinn KE, Catanach A, Fullerton C, Li D, Meiyalaghan S, Nieuwenhuizen N, Read N, Prakash R, Hunter D, Zhang H, McKenzie M, Knäbel M, Harris A, Allan AC, Gleave A, Chen A, Janssen BJ, Plunkett B, Ampomah-Dwamena C, Voogd C, Leif D, Lafferty D, Souleyre EJF, Varkonyi-Gasic E, Gambi F, Hanley J, Yao J-L, Cheung J, David KM, Warren B, Marsh K, Snowden KC, Lin-Wang K, Brian L, Martinez-Sanchez M, Wang M, Ileperuma N, Macnee N, Campin R, McAtee P, Drummond RSM, Espley RV, Ireland HS, Wu R, Atkinson RG, Karunairetnam S, Bulley S, Chunkath S, Hanley Z, Storey R, Thrimawithana AH, Thomson S, David C, Testolin R, Huang H, Hellens RP, Schaffer RJ (2018) A manually annotated *Actinidia chinensis* var. *chinensis* (kiwifruit) genome highlights the challenges associated with draft genomes and gene prediction in plants. BMC Genomics **19:** 257

Varkonyi-Gasic E, Moss SMA, Voogd C, Wang T, Putterill J, Hellens RP (2013) Homologs of *FT*, *CEN* and *FD* respond to developmental and environmental signals affecting growth and flowering in the perennial vine kiwifruit. New Phytologist **198:** 732-746

Varkonyi-Gasic E, Wang T, Cooney J, Jeon S, Voogd C, Douglas MJ, Pilkington SM, Akagi T, Allan AC (2021) *Shy Girl*, a kiwifruit suppressor of feminization, restricts gynoecium development via regulation of cytokinin metabolism and signalling. New Phytologist **230:** 1461-1475

Varkonyi-Gasic E, Wang T, Voogd C, Jeon S, Drummond RSM, Gleave AP, Allan AC (2019) Mutagenesis of kiwifruit *CENTRORADIALIS*-like genes transforms a climbing woody perennial with long juvenility and axillary flowering into a compact plant with rapid terminal flowering. Plant Biotechnology Journal **17:** 869-880

Voogd C, Brian LA, Wang T, Allan AC, Varkonyi-Gasic E (2017) Three *FT* and multiple *CEN* and *BFT* genes regulate maturity, flowering, and vegetative phenology in kiwifruit. Journal of Experimental Botany **68:** 1539-1553

Voogd C, Brian LA, Wu R, Wang T, Allan AC, Varkonyi-Gasic E (2022) A MADS-box gene with similarity to *FLC* is induced by cold and correlated with epigenetic changes to control budbreak in kiwifruit. New Phytol **233:** 2111-2126
